# Fit & Strong! Promotes Physical Activity and Well-Being in Older Cancer Survivors

**DOI:** 10.3389/fpubh.2014.00171

**Published:** 2015-04-27

**Authors:** Jana Reynolds, Lorie Thibodeaux, Luohua Jiang, Kevin Francis, Angie Hochhalter

**Affiliations:** ^1^Baylor Scott & White Health, Temple, TX, USA; ^2^Baylor Charles A. Sammons Cancer Center, Baylor University Medical Center at Dallas, Dallas, TX, USA; ^3^Seton Medical Center, Austin, TX, USA; ^4^School of Rural Public Health, Texas A&M Health Science Center, College Station, TX, USA

**Keywords:** evidenced based intervention, older cancer survivors, physical activity, exercise, cancer survivorship

## Abstract

**Introduction:**

Physical activity reduces fatigue and depression while improving quality of life in cancer survivors. Exercise is generally considered safe and is recommended to survivors of all ages. Despite the high prevalence of cancer in the elderly, few studies address physical activity interventions targeting this older population. Fit & Strong! is an evidence-based physical activity program shown to improve level of physical activity, exercise-self-efficacy, and mood in older adults with osteoarthritis. This study tests the feasibility and short-term impact of the Fit & Strong! exercise program adapted for older cancer survivors.

**Methods:**

Participants were cancer survivors at least 50 years of age who were not on active treatment with intravenous chemotherapy or radiation. They participated in the 8-week Fit & Strong! program, which included three 90-min sessions per week; 60 min of group physical activity and 30 min of education. Education on osteoarthritis was removed from the Fit & Strong! program and replaced with relevant topics on cancer survivorship issues. Feasibility was measured by the ability to recruit and retain older cancer survivors. Pre and post-intervention surveys evaluated the effect of the intervention on physical activity and quality of life.

**Results:**

The study enrolled 72 cancer survivors to participate in an 8-week exercise program. The mean age of participants was 70. Over two-thirds (68%) of participants completed the program and with a mean attendance rate of 75% (18 of 24 sessions). No safety issues occurred. Improvements from baseline to post-intervention were observed for self-reported minutes of physical activity per week, self-efficacy for aerobic exercise, and symptoms related to depression and anxiety.

**Conclusion:**

This study was successful in recruiting and retaining a population of older cancer survivors to participate in a group exercise program. Significant improvement in level of physical activity and mood suggests this evidence-based physical activity intervention can be adapted to promote health benefits in cancer survivors. Additional studies are necessary to confirm efficacy and assess long-term benefits.

## Introduction

With early cancer detection and greater availability of curative therapy, 64% of cancer survivors in the United States are living five or more years after cancer diagnosis (1, 2). As the number of long-term survivors continues to increase, so has the recognition of negative late and long-term health effects of cancer and cancer treatment (2, 3). It is well documented that once cancer survivors complete their initial treatment, many face persistent fatigue, depression, fear of recurrence, and long-term physical effects of treatment (3–7). Thus, finding ways to combat these long-term health effects in cancer survivors is of paramount importance.

One way to address these long-term effects of cancer and cancer treatment is through increased physical activity. Physical activity in cancer survivors reduces fatigue and depression while improving quality of life (8–12), and at the same time has been shown to be safe in this population (13). It is recommended that cancer survivors of all ages participate in a combination of strength training and moderate aerobic exercise (such as brisk walking) for at least 150 min per week, or to the best of their physical ability. These guidelines are similar to those recommended for the general population (13, 14). Additionally, results from observational studies suggest that participation in physical activity before and/or after diagnoses of certain cancers may serve as a potential preventive measure against recurrence and mortality (4, 15–19).

In the United States, nearly 90% of cancer survivors are aged 50 and older (88%), with persons aged 70 and older accounting for almost half (46%) of all cancer survivors (2). Despite the frequency of cancer in the elderly, the majority of studies targeting physical activity in cancer survivors either exclude or do not achieve high levels of participation from older cancer survivors (20, 21). Given the prevalence of cancer in the older population and lack of evidence-based physical activity programs engaging this population, this study aims to test a group exercise intervention targeting older cancer survivors.

This study chose Fit & Strong!, an evidence-based physical activity program for older adults with osteoarthritis, to adapt to a population of older cancer survivors. The program is a combination of group exercise and education/support. In randomized controlled trials, Fit & Strong! significantly increased participation in physical activity while decreasing levels of anxiety and depression and reducing symptoms of osteoarthritis in adults older than 60 (22–24). We chose to use the Fit & Strong! program because of its relative low cost and ease of reproducibility. Additionally, the program adapts to the abilities of individual participants and thus would be reasonable to implement in a population of older cancer survivors with differing capacities for exercise. The program includes basic education on exercise with the goal of sustainability through a continued home-based program (22–24). Our adaptation replaces the osteoarthritis specific educational curriculum with education addressing important cancer survivorship issues.

The primary aim of the study was to evaluate the feasibility of recruiting and retaining older survivors to participate in an 8-week group exercise intervention and education program. The secondary aim was to test the short-term impact of the Fit & Strong! intervention on self-reported physical activity, self-efficacy for exercise, and quality of life.

## Materials and Methods

### Participants

Participants were eligible for this study if they (1) were 50 years of age or older, (2) had a previous diagnosis of cancer, (3) were not on active cancer treatment such as chemotherapy or radiation, and (4) were self-reported able to engage in light-to-moderate physical activity. Although the intention was to target older cancer survivors (i.e., 65 years of age and up), we chose to allow participants 50 years and older. This age allowance was in recognition that some younger patients with lower functional status, either at baseline or due to cancer or treatment effects, similarly might benefit from the intervention. There was no restriction on type of cancer or years since cancer diagnosis for patient eligibility. Individuals taking oral hormonal or biologic treatments for their cancer were allowed to participate at the discretion of the physician investigator (Jana Reynolds). This study was approved by the Institutional Review Board of Scott & White Healthcare.

### Recruitment

Participants were recruited by advertisement put in the local newspaper and flyers placed at senior centers, community cancer survivorship events, and local oncology clinics. The mode of recruitment that generated the most interest in our program, located in rural Central Texas, was newspaper advertisement. Interested individuals contacted the program coordinator and were screened by phone survey to determine whether they met eligibility criteria. Those who met enrollment criteria were invited to participate in the study by enrolling in one of five courses. Participants gave their consent and official enrollment occurred during the first session of each course.

### Course setting

The intervention was offered as an 8-week exercise course with three 90-min sessions per week, for a total of 24 sessions. Approximately 60 min of each session was dedicated to physical activity, and 30 min was dedicated to education. Each course was conducted in a group setting with a goal class size of 8–20 participants. A total of five courses were offered between January 2013 and August 2013. The first course was conducted in a large conference room in a medical office building. Due a higher than anticipated number of participants, the subsequent four courses were offered in a larger aerobics room at a local health center.

### Safety

All participants were encouraged to consider consulting with a physician prior to beginning of the program. During the eligibility screening phone calls, participants were screened for the presence of specific medical conditions including recent joint surgery or current rehabilitation for joint surgery, known cancer metastases to bone (indicating higher risk of fracture), or history of cardiac disease. Interested participants with these or any other health-related concerns were required to contact their physician to discuss participation prior to enrollment. They were prompted to describe the course as “mild to moderate physical activity that includes walking and light weight lifting,” and ask if there were particular types of activities they should avoid. All course instructors were certified in Basic Life Support.

### Adaptation of Fit & Strong! exercise intervention

Prior to enrolling participants, a license to conduct the Fit & Strong! program was obtained through the Fit & Strong! program office (Institute for Health Research and Policy at the University of Illinois at Chicago). Additionally, our two instructors completed a Fit & Strong! Master Training Program. Fit & Strong! Master Training instruction provided 8 h of education on topics including appropriate types of exercises for older adults and how to implement Fit & Strong! in the community setting. The program supplied instructional manuals for the instructors to follow when facilitating Fit & Strong! courses. In addition to this training, our two instructors held certifications in Chronic Disease Self-Management (Stanford CDSMP). They were experienced in leading group discussion of health behaviors among adults, but our instructors had limited experience leading group exercise activities. At least one of our two trained instructors and one assistant facilitated each 90 min session.

Participants had exercise equipment available as recommended by Fit & Strong! This equipment included resistance bands for arm exercises, 10 pound adjustable ankle weights for leg exercises, and mats for floor-based exercises. Chairs were available for sitting exercises or for those who required modification to their exercise program. Unique to our study, those participating in courses at the local health center had the option to use exercise machines, such as treadmills and stationary bikes, for the aerobics portion of the class.

Fifty to 60 min of each 90-min class session was dedicated to aerobic and strength-training activities. The complete instructor-led exercise routine consisted of a 5- to 15-min warm-up with stretching, 15–20 min of an aerobic activity, 15–20 min of resistance training, and a 5-min cool-down session. Resistance-training exercises followed those recommended in the Fit & Strong! instructor handbook (i.e., leg lifts while seated in a chair). The aerobic component consisted of sustained walking and a low-impact aerobics routine created by our instructors. Each session used this complete exercise routine in the same sequence. Fit & Strong! trained instructors monitored participants and made adaptations of exercises as needed to match participant abilities.

Thirty minutes of each 90-min class session was dedicated to education designed to increase self-efficacy for exercise and exercise adherence (23, 24). The exercise education curriculum was taught by our instructors and a Fit & Strong! manual was provided to each participant for reference during each class. Information on the components of an exercise program and exercise safety was presented (Table 1). Participants engaged in group problem-solving activities and set physical activity goals. Two educational sessions specific to osteoarthritis were removed from the original Fit & Strong! curriculum for our program because they were not applicable to all cancer survivors.

**Table 1:** **Sample course curriculum**.

Session	Fit & Strong! exercise curriculum	Cancer survivorship curriculum
	Source: Fit & Strong participant manual (http://www.fitandstrong.org/index.html)	Source: NCI facing forward: life after cancel treatment (25)
1	Introduction, consent, and baseline survey	
2	Introduction to Fit & Strong (Ch 1)	Definition of survivorship (preface) and finding a new “Normal” (p. 1)
3	Benefits and barriers of exercise (Ch 2)	Follow-up medical care (pp. 2–5)
4	What to wear (Ch 3)	
5	Pain and exercise modifications (Ch 6)	Creating a wellness plan (pp. 5–11)
6	Warm-up exercises (Ch 7)	Services and community resources (pp. 12–13)
7	Stretching (Ch 8)	Nutrition for cancer survivors^a^
8	Aerobic exercise (Ch 9)	
9		Treatment effects, Part I: fatigue, memory, and concentration (pp. 15–19)
10	Walking (Ch 10)	Treatment effects, Part II: pain and physical changes (pp. 20–31)
11	Strengthening exercise (Ch 11)	
12	Resistance training (Ch 12)	Managing your feelings: stress, depression, anxiety (pp. 37–45)
13	Cool-down exercises (Ch 13)	Finding a meaning (pp. 46–48) and making a difference after Cancer^b^
14	Posture and bone health (Ch 14)	
15	Fall prevention (Ch 15)	Social and work relationships (pp. 49–55)
16	Setting goals (Ch 16)	
17	Other ways to do exercise (Ch17)	Learning to relax: instructor guided relaxation exercise no. 1 (p. 60)
18	Lifestyle changes (Ch 18)	
19	Exercise: a world of options (Ch 19)	Support for caregivers^c^
20	Getting past barriers to exercise (Ch 20)	
21	Diet and exercise (Ch 21)	
22	Stress management (Ch 22)	Learning to relax: instructor guided relaxation exercise no. 2 (pp. 60–61)
23	Maintaining an active lifestyle (Ch 23)	Feedback session on survivorship component
24	Putting it all together (Ch 24) and survey	

***Each 90 min session included 60 min of exercise and 30 min of education. A sample schedule for the educational component is listed above. All chapters/page numbers refer to the source listed in heading unless otherwise noted***.

*^a^National Cancer Institute: “Eating hints; Before, During, and After Cancer Treatment” pp.44–45 (26)*.

*^b^National Cancer Institute: “Facing Forward. Making a difference in cancer” (27)*.

*^c^National Cancer Institute: “Facing Forward: when someone you love has completed cancer treatment” (28)*.

### Survivorship education component of the Fit & Strong! program

The adaptation of the Fit & Strong! program tested in this study replaced the original education on osteoarthritis with cancer-related topics. The content for cancer survivorship education came from the National Cancer Institute’s *Facing Forward* series along with additional materials from the National Cancer Institute (25–29). Topics included the long-term effects of cancer treatment, self-management of the long-term physical and psychosocial effects of cancer and cancer treatment, nutrition for cancer survivors, support for the caregiver, seeking follow-up medical care, and ways to make a difference after cancer (Table 1). Participants were provided copies of the printed materials to reference in class, and if desired, to keep for future reference.

Trained Fit & Strong! instructors incorporated the cancer survivorship materials during the 30 min educational sessions. They presented information from the handouts and then facilitated the group discussion. Clinicians specializing in the cancer care field were invited to teach the cancer-specific curriculum in two to four sessions for each course (Jana Reynolds, Kevin Francis). Participants with cancer-specific questions beyond the scope of the course materials were encouraged to ask their oncologist or health care provider.

### Outcome measures

The primary aim of feasibility of Fit & Strong! for older cancer survivors was measured by the course completion rate. Participants were considered to have successfully completed the study if they filled out a survey at baseline and course completion, pre-defined as within 1 week of the 24th (final) session. Instructors documented attendance and calculated the total number of sessions attended by each participant. Self-reported demographics and disease characteristics were obtained at baseline to describe the population and identified potential characteristics of likely participants for similar studies. These data included gender, age, weight, height, ethnicity, race, marital status, employment status, type of cancer, time since treatment completion, and whether one considered if they are living with active cancer (yes/no).

The secondary aim of the study was to test the short-term impact of the intervention on exercise and quality of life. This aim was measured by changes in baseline and post-intervention surveys comparing minutes of physical activity, self-efficacy for exercise, and cancer-related quality of life. The surveys are as follows:

#### Minutes of physical activity

Participants reported the number of days in the past 7 days and they did moderate to strenuous exercise. They also reported how many minutes, on average, they exercised per day. Physical activity time per week was calculated by multiplying the reported days by the reported minutes, similar to the original Fit & Strong! study (22–24).

#### Self-efficacy for exercise

Self-efficacy for exercise was measured on a three item scale developed Lorig and colleagues (30). Participants reported their confidence to do frequent aerobic exercise, frequent strengthening exercise, and confidence to participate in exercise without making their symptoms (of chronic disease) worse. This was reported on a 10 point scale of “not at all confident” (score of 1) to “totally confident” (score of 10). A calculation of the mean rating across the three questions determined the score on this measure. This measure showed improvement in exercise-self-efficacy at 2, 6, and 12 months for participants with osteoarthritis in the original Fit & Strong! intervention (22–24).

#### Cancer-related quality of life

Participants completed the quality of life in adult cancer survivors (QLACS) survey, a 47-item questionnaire with five cancer-specific and seven generic domains. This survey captures issues affecting long-term cancer survivors rather than acute cancer or cancer treatment-related effects. Cancer-related domains of the survey include concerns with appearance, financial problems, distress over recurrence, family-related distress, and benefits of cancer. Generic domains include negative feelings, positive feelings, cognitive problems, physical pain, fatigue, and social avoidance. The scores of each domain and a summary score of the cancer-related (seven items) and generic domains (four items, benefits of cancer not included in the summary score) were reported (31).

At course completion, a course evaluation survey captured the participant’s satisfaction with the exercise and cancer-specific portions of the program. It allowed participants to provide suggestions for improvement. The intention of this survey was to provide feedback for future studies.

### Statistical analysis

Participant characteristics at baseline and the study completion rate used descriptive statistics. The impact of the intervention on exercise efficacy, physical activity, and quality of life was assessed using paired *t*-tests. Significance was defined as *p* ≤ 0.05.

## Results

Seventy-two (72) cancer survivors participated in one of five courses offered as part of this study, with an average of 14 participants per 8-week course. The mean age of participants was 70.4 (±13.3) years. Forty-nine of the 72 participants completed the course, for a 68% retention rate. The mean number of sessions attended by those who completed the course was 18 out of 24 (75%).

Participant characteristics are illustrated in Table 2. The majority of participants were female (82%). The average BMI at baseline was 29.08 (±6.79), with 40% of participants considered obese (BMI 30 or greater). Patients with 18 types of cancer were represented in the study, with the majority (52%) of participants reporting a prior breast cancer diagnosis. Almost half the participants (46%) had been diagnosed and completed cancer treatment at least 5 years prior, with a median time since treatment of 7 years. Though not on active intravenous chemotherapy or radiation per study protocol, six participants (8%) considered themselves to have active cancer during the study.

**Table 2:** **Participant demographics and cancer history**.

	*N* (%)
Age	
<70	37 (52.11)
≥70	34 (47.89)
Sex	
Male	13 (18.06)
Female	59 (81.94)
Race	
White	61 (89.71)
Other	7 (10.29)
Martial Status	
Married	37 (54.41)
Not married	31 (45.59)
Employment Status	
Employed	8 (12.12)
Not employed	58 (87.88)
BMI	
<20	3 (4.41)
20–25	20 (29.41)
25–30	18 (26.47)
30–35	17 (25.00)
35+	10 (14.71)
Type of Cancer	
Breast	37 (52.11)
Colon	5 (7.04)
Prostate	5 (7.04)
Lung	4 (5.63)
Other^a^	20 (28.17)
Time since completion of cancer treatment	
<1 year	14 (20.90)
1–5 years	22 (32.84)
5+ years	31 (46.27)
Participants who consider themselves to have active cancer	
No	66 (91.67)
Yes	6 (8.33)

*^a^Hodgkin’s lymphoma, non-Hodgkin’s lymphoma, chronic leukemia, multiple myeloma, adenoid cystic cancer, gastrointestinal stromal tumor, bladder, kidney, pancreatic, thyroid, ovarian, uterine, cervical, and vulvar cancer*.

Participants significantly increased their weekly total minutes of moderate to strenuous exercise from baseline to post-intervention (94.1 vs. 131.5 min, *p* = 0.0005). Their overall self-efficacy for exercise (i.e., average of the three self-efficacy for exercise items) did not differ from baseline to post-intervention (*p* = 0.0964). However, in the measure of self-efficacy for doing for aerobic exercise regularly, participants showed significant improvement in their ratings from baseline to post-intervention (*M*_Baseline_ = 7.94, *M*_Post-intervention_ = 8.85; *p* = 0.05) (Table 3).

**Table 3:** **Intervention impact on exercise efficacy and total minutes of physical activity by paired *t*-test**.

		Baseline (*n* = 72)	Post (*n* = 49)	Paired change (*n* = 49)	*t*	*p*
		Mean (±SD)	Mean (±SD)	Mean (±SD)	
1.	Confidence to do strength and flexibility exercises 3–4 times a week	8.68 (±1.75)	9.19 (±1.50)	0.45 (±1.86)	1.64	0.1069
2.	Confidence to do aerobic exercises 3–4 times a week	7.94 (±2.45)	8.85 (±1.96)	0.69 (±2.37)	2.01	**0.0503**
3.	Confidence to exercise without making symptoms (of chronic disease) worse	8.51 (±1.98)	8.60 (±2.20)	0.10 (±2.26)	0.32	0.7511
Overall self-efficacy for exercise (mean of 1–3)		8.39 (±1.73)	8.88 (±1.67)	0.41 (±1.69)	1.70	0.0964
Total minutes of physical activity		94.10 (±87.02)	131.51 (±91.01)	42.22 (±73.80)	3.79	**0.0005**

*Bold text indicates statistically significant values (p ≤ 0.05)*.

Participant’s scores on the generic and cancer-specific subscale of the QLACS survey did not differ from baseline to post-intervention (*p* = 0.0770 and *p* = 0.9303, respectively). Nonetheless, an improvement in scores on the negative feeling domain (questions related to anxiety and depression) was observed from baseline to post-intervention (*p* = 0.0198) (Table 4).

**Table 4:** **Intervention impact on Quality of Life (QLACS scale) by paired *t*-test**.

	Baseline (*n* =72)	Post (*n* =49)	Paired change (*n* =49)	*t*	*p*-value
	Mean (±SD)	Mean (±SD)	Mean (±SD)		
**GENERIC QOL**					
Negative feelings^a^	10.18 (±4.40)	9.22 (±3.62)	−1.27 (±3.17)	−2.44	**0.0198**
Positive feelings	10.26 (±4.69)	10.32 (±4.72)	−0.72 (±3.85)	−1.17	0.2509
Cognitive problems	10.47 (±4.02)	10.43 (±3.53)	0.04 (±3.11)	0.10	0.9241
Sexual problems	11.83 (±6.49)	10.29 (±5.55)	−1.59 (±4.81)	−1.78	0.0864
Energy/fatigue	14.81 (±2.81)	14.67 (±4.21)	0.15 (±4.83)	0.21	0.8319
Pain	11.66 (±5.74)	11.00 (±5.52)	−0.54 (±4.95)	−0.69	0.4912
Social avoidance	9.11 (±5.19)	8.57 (±3.73)	0.12 (±3.14)	0.25	0.8049
Generic summary score	77.42 (±24.99)	73.03 (±18.56)	−8.52 (±22.03)	−1.86	0.0770
**CANCER-SPECIFIC QOL**					
Financial problems	7.00 (±4.75)	6.48 (±4.78)	0.30 (±2.46)	0.80	0.4308
Benefits	19.24 (±6.24)	19.49 (±6.36)	0.51 (±4.38)	0.77	0.4483
Distress-family	9.43 (±5.61)	8.80 (±5.00)	−0.18 (±3.92)	−0.30	0.7621
Appearance	8.80 (±5.70)	8.48 (±4.82)	0.04 (±3.91)	0.08	0.9395
Distress-recurrence	12.79 (±6.33)	12.91 (±6.09)	−0.21 (±3.26)	−0.42	0.6755
Cancer-specific summary score	37.95 (±16.88)	36.89 (±14.81)	−0.12 (±8.87)	−0.09	0.9303

*^a^Questions in the negative feeling domain (1) bothered by mood swings, (2) felt blue or depressed, (3) worried about little things, and (4) felt anxious*.

*Bold text indicates statistically significant values (p ≤ 0.05)*.

In a post-intervention survey of the utility of the course and the cancer-specific education, 79% reported learning something helpful about cancer survivorship that they did not know before starting our course. Sixty-eight percent of participants reported sharing the information on support for caregivers with a friend or a family member. Some enjoyed meeting others with similar experiences and took pleasure in sharing information. All respondents stated that they would recommend the course to another cancer survivor.

One of the survivors thought the survivorship discussions were too emotional. Another reported feeling uncomfortable during voluntary group discussion, preferring more privacy regarding cancer survivorship issues. Some desired a longer exercise course or to be allowed to repeat the course again. Though participants were encouraged to set goals and create a plan for sustained exercise beyond the program, many wanted to continue within the current group setting.

## Discussion

Despite the known physical and emotional benefits of exercise in cancer survivors, the majority of studies targeting physical activity in this population either exclude or do not achieve high levels of participation from older cancer survivors (20, 21). Our study was successful in recruiting a population of older cancer survivors with a mean age of 70. The 68% retention rate and 75% session attendance rate is indicative of an intervention individuals were willing to engage in over time. These results support the feasibility of recruiting and retaining older cancer survivors to participate in an 8-week group exercise intervention and education program.

Our study utilized Fit & Strong!, an evidence-based physical activity intervention for older adults with osteoarthritis, as it previously showed long-term physical activity benefits in older adults with a mean age of 73 (23, 24). Hughes and colleagues observed similar retention rate (72%) and attendance (79% of sessions) in their original Fit & Strong! for osteoarthritis study (23). Our study kept the same physical activity content and adapted the educational component of Fit & Strong! by replacing osteoarthritis education with education on common issues facing cancer survivors. No major safety issues were reported.

Participants in our study showed improvement in level of physical activity and mood, supporting the short-term efficacy of Fit & Strong! when adapted to a population of older cancer survivors. Participants successfully increased their self-reported weekly minutes of physical activity from 94.1 minutes at the beginning of the study to 131.5 minutes at the end of the 8-week intervention (Table 3). Participants showed significant improvement in the negative feeling domain of the cancer-related quality of life assessment (QLACS), though not in overall quality of life (Table 4). The questions in the negative feeling domain address depression and anxiety, which are reported more commonly in cancer survivors and should be a specific measure in future studies (3, 4).

Participants also improved exercise-self-efficacy specific to aerobic activity; however, no changes were observed in overall exercise-self-efficacy (Table 3). This is in contrast to the findings of the original Fit & Strong! intervention in which participants with osteoarthritis showed improvement on the overall exercise-self-efficacy scale at 2, 6, and 12 months (22, 23). One explanation of the variation between the studies is that the self-efficacy scale is more specific to persons with symptoms of osteoarthritis. The item “confidence to do exercise without making symptoms of chronic disease worse” may be more relevant to osteoarthritis pain symptoms rather than a population of cancer survivors with a wide variation of chronic symptoms. A self-efficacy scale examining perceived ability to do exercise without a focus on symptomatology of chronic disease may be more appropriate for cancer survivors.

This study had several limitations. First, this study was not designed to test long-term effects of the intervention on physical activity, self-efficacy, or cancer-related quality of life. Studies of long-term efficacy and sustained benefits will be necessary to establish whether this program is likely to have meaningful impact on outcomes for cancer survivors beyond the 8-week intervention. Second, the study did not measure the effects of the intervention on actual physical health or function; outcomes were limited to self-reported measures. Future studies should consider tests of the intervention effects using direct measures of physical health and function.

Additionally, participants did not meet the 150 minutes of physical activity per week as recommended in the guidelines (13, 14). Though it is reasonable for capable participants to strive to this goal, it may not be necessary to gain benefits of exercise. In a separate study of older cancer survivors, an increase in minutes of physical activity over baseline but to less than a total of 150 minutes per week still showed measurable functional and health-related benefits (32).

Despite the limitations of this study, the majority of the feedback on the program was positive. Most participants indicated that they would recommend a similar course to other survivors. Many participants expressed appreciation for meeting other cancer survivors and sharing experiences. Most participants in our study were female (82%), suggesting this type of group intervention may be of particular interest to females. Almost half of the participants were at least 5 years post-cancer treatment, indicating older cancer survivors are interested in a cancer-related exercise program long after they finish their treatment (Table 2). Cancer survivors undergoing therapy were excluded from the protocol for the purpose of keeping baseline characteristics similar and to minimize conflicts between class times and cancer treatment schedules. Given that exercise is safe for most patients while undergoing treatment, it would be reasonable to include them in future programs (20).

Anecdotally, many participants reported a desire to continue the course indefinitely as their primary exercise program. The Fit & Strong! intervention focuses primarily on initiating an exercise routine that could be sustained in one’s home after course completion. The original version was not designed to continue in a group setting. Cancer survivors may benefit from an additional adaptation that helps participants find appropriate community-based group exercise programs with social support similar to the Fit & Strong! program. It would also be reasonable to consider monthly maintenance classes open to all graduates to help inspire and refocus exercise goals for long-term sustainability of benefits.

This paper is included in the Research Topic, “Evidence-Based Programming for Older Adults.” This Research Topic received partial funding from multiple government and private organizations/agencies; however, the views, findings, and conclusions in these articles are those of the authors and do not necessarily represent the official position of these organizations/agencies. All papers published in the Research Topic received peer review from members of the Frontiers in Public Health (Public Health Education and Promotion section) panel of Review Editors. Because this Research Topic represents work closely associated with a nationwide evidence-based movement in the US, many of the authors and/or Review Editors may have worked together previously in some fashion. Review Editors were purposively selected based on their expertise with evaluation and/or evidence-based programming for older adults. Review Editors were independent of named authors on any given article published in this volume.

## Conclusion

Results of this pilot study support the feasibility of implementing an 8-week exercise intervention for older cancer survivors. Short-term efficacy of the Fit & Strong! program was noted from baseline to the end of the 8-week intervention by increases in minutes of physical activity, increased self-efficacy for aerobic exercise, and decreased negative feelings in the quality of life (QLACS) scale. Tests of efficacy and effectiveness over time are needed to determine the utility of this intervention as a program to promote sustained physical activity among older cancer survivors and support long-term health outcomes.

## Conflict of Interest Statement

The authors declare that the research was conducted in the absence of any commercial or financial relationships that could be construed as a potential conflict of interest.

*This paper is included in the Research Topic, “Evidence-Based Programming for Older Adults.” This Research Topic received partial funding from multiple government and private organizations/agencies; however, the views, findings, and conclusions in these articles are those of the authors and do not necessarily represent the official position of these organizations/agencies. All papers published in the Research Topic received peer review from members of the Frontiers in Public Health (Public Health Education and Promotion section) panel of Review Editors. Because this Research Topic represents work closely associated with a nationwide evidence-based movement in the US, many of the authors and/or Review Editors may have worked together previously in some fashion. Review Editors were purposively selected based on their expertise with evaluation and/or evidence-based programming for older adults. Review Editors were independent of named authors on any given article published in this volume*.
